# Ginsenoside Rh1 potentiates dexamethasone’s anti-inflammatory effects for chronic inflammatory disease by reversing dexamethasone-induced resistance

**DOI:** 10.1186/ar4556

**Published:** 2014-05-01

**Authors:** Jun Li, Juan Du, Dong Liu, Binbin Cheng, Fanfu Fang, Li Weng, Chen Wang, Changquan Ling

**Affiliations:** 1 Department of Traditional Chinese Medicine, Changhai Hospital, Second Military Medical University, 168 Changhai Road, Shanghai 200433, P.R. China

## Abstract

**Introduction:**

Acquired resistance to glucocorticoids constitutes a major clinical challenge, often overlooked in the search for compounds to improve the effect of classic steroids. We sought to unravel how a plant-original compound, ginsenoside Rh1, potentiates dexmethasone (DEX)’s potential anti-inflammation properties.

**Methods:**

Ginsenoside Rh1 combined with DEX was applied in a short-term and long-term treatment protocol for inflammation. Its potential mechanism on anti-inflammation was explored. In addition, the effect of Rh1 on the side-effect induced by DEX was studied. Furthermore, the *in vivo* anti-inflammatory effects of Rh1 combined with DEX were evaluated in a collagen-induced arthritis (CIA) mice model.

**Results:**

Ginsenoside Rh1 potentiates DEX’s anti-inflammatory effects even after prolonged DEX treatment. Rh1 could improve the glucocorticoid receptor (GR)’s transrepression on nuclear factor kappa B (NF-κB) and transactivation on dual specificity protein phosphatase 1 (DUSP1), which is responsible for DEX’s anti-inflammatory effects. Parallel Western blot assay and radioligand binding analysis revealed that Rh1 could increase the expression and binding of GR. This is in sharp contrast to DEX alone, showing a direct link among prolonged treatment, decreasing GR and the abolishment of anti-inflammation. Interestingly, Rh1 does not enhance the transactivation of glucocorticoid-responsive elements (GRE) driven genes - *gluconeogenic enzyme glucose-6-phosphatase* (G6P) and *phosphoenolpyruvate carboxykinasee phosphatase* (PEPCK) in primary mouse hepatocytes, a mechanism partly held accountable for the metabolic side-effects. Similar results were found in CIA mice.

**Conclusion:**

Rh1 could potentiate DEX’s anti-inflammatory effects and does not cause a hyperglycemic side effect. Ginsenoside Rh1 combined with DEX may be a promising candidate treatment option for chronic inflammatory diseases in need of long-term immunosuppression therapies.

## Introduction

Glucocorticoids (GCs) are still the cornerstone drugs used in treatment protocols of a wide range of inflammatory and immune disorders. However, long-term and/or high-dose GC administration is commonly associated with adverse side effects, such as hyperglycemia, weight gain, osteoporosis, depression and decreased immunological function. Furthermore, patients on glucocorticoids can develop reduced glucocorticoid sensitivity and even resistance. It has been reported that approximately 30% of patients failed to respond to even high doses of glucocorticoids [1,2].

Different molecular mechanisms have been responsible for the phenomenon of acquired glucocorticoid resistance, including reduced expression of the glucocorticoid receptor (GR), altered affinity of GR for the ligand, reduced ability of GR to bind DNA or increased expression of inflammatory transcription factors, such as AP-1, that compete for DNA binding [3-5].

Current research is focused on finding compounds with similar anti-inflammatory potency of the standard GCs but with reduced side effects [6-9]. Nevertheless, it is currently unclear whether simply dissociating activation from repression of GR in a ligand will result in a beneficial therapeutic profile. Actually, the powerful anti-inflammatory effect of GCs is complex, and likely due to both repression of a large number of pro-inflammatory cytokines and mediators, as well as activation of anti-inflammatory genes, such as *IL-10*, *IL-4*, *TGF-β*, *DUSP1* and *Annexin A1*[10-12]. As a result, novel compounds that lack a capacity for steroid-inducible genes may in fact show lessened anti-inflammatory effects [13-15]. In fact, the regulation of GR may be another efficient strategy for restoring glucocorticoid resistance.

Over the past years, our team has had a continued interest in the regulation of GR with Chinese medicinal herbs [16,17]. We have demonstrated that the main extracts of ginseng, ginsenosides (GSS), which is one of the derivatives of triterpenoid dammarane consisting of 30 carbon atoms, could partially reverse dexmethasone (DEX)-induced down-regulation of GR *in vitro* and *in vivo*[18]. But there are many ingredients in GSS. Ginsenoside Rh1 is one of the major one of these.

We thus studied the effect of ginsenoside Rh1 on GR and how this compound improved DEX’s anti-inflammatory potential by regulating GR after prolonged DEX treatment. In addition, we unraveled the potential of Rh1 on GCs-induced hyperglycemia. Also, we evaluated the anti-inflammatory effects of Rh1 combined with DEX *in vivo*.

## Materials and methods

### Cytokines and reagents

Recombinant murine TNF-α was purchased from R&D. Ginsenoside Rh1 was supplied by Shanghai Dongfang Pharmaceutical (Shanghai, China). Rabbit polyclonal Ab to GR was obtained from Abcam (Cambridge, MA, USA). Phospho-IκBa (Ser32) rabbit mAb, phospho-NF-κB p65 (Ser536), phospho-p38, IκBa (44D4) rabbit mAb, NF-κB p65 and p38 Abs were supplied by Cell Signaling Technology (Danvers, MA, USA).

### Cell culture

The murine macrophage RAW264.7 cells were purchased from American Type Culture Collection (Manassas, VA, USA) and maintained in Dulbecco’s modified Eagle’s medium (DMEM) (Invitrogen, Carlsbad, CA, USA) supplemented with 10% FBS, 100 U/ml penicillin, and 0.1 mg/ml streptomycin in a 5% CO_2_ humidified incubator at 37°C. The primary mouse hepatocytes were isolated and cultured as described in [19].

### Western blot analysis

Cells were seeded in six-well plates. Total protein, cytoplasmic and nuclear proteins were extracted as described previously [20].

### Real-time PCR

RT-PCR was performed as described in [21]. Primer sets for interleukin-6 (IL-6), IL-17, matrix matalloproteinase-1 (MMP-1), tumor necrosis factor-alpha (TNF-α), phosphoenolpyruvate carboxykinasee phosphatase (PEPCK), gluconeogenic enzyme glucose-6-phosphatase (G6P), dual specificity protein phosphatase 1 (DUSP-1), GR and glyceraldehyde 3-phosphate dehydrogenase (GAPDH) are available upon request.

### ELISA

IL-6 and IL-17 levels were determined by ELISA kits according to the manufacturer’s instructions (Bender MedSystems, Vienna, Austria).

### Radioligand binding analysis for GR

Radioligand binding analysis was performed as described in [18].

### Transient transfection and luciferase assay

After RAW264.7 cells reached 90 to 95% confluence in 24-well dishes, they were transfected using Lipofectamine 2000 (Invitrogen) with 0.6 mg/ml p-GR-Luc and 0.08 mg/ml p-Renilla luciferase-thymidine kinase for normalization in serum-free medium. Four hours later, the medium was replaced with DMEM containing 10% FBS. Twenty-four hours post-transfection, cells were pretreated for 1 h with or without 10 μM Rh1, after which DEX (1 μM) was added for 24 h. Then the reporter gene activity was measured according to the recommendation of the manufacturer (Promega, Madison, WI, USA). Results were normalized by thymidine kinase reporter activity.

### Collagen-induced arthritis mouse model

Male DBA/1 mice (six to eight weeks old) were purchased from the Second Military Medical University Laboratory Animal Center (Shanghai, China). The induction of collagen-induced arthritis (CIA) in DBA/1 mice is described in [22,23]. Once arthritis was evident, the animals were randomized in one of the following treatment groups with eight mice per group: vehicle, Rh1 and DEX, and DEX by intraperitoneal (i.p.) therapy for 10 d. An additional eight normal mice were as blank group. The clinical severity of arthritic paws was graded according to standard evaluation procedures. For more details refer to [22].

All procedures involving animals were performed in accordance with the European Communities Council Directive of November 24, 1986 (86/609 EEC) and approved by the Ethics Committee of Changhai Hospital.

### Blood glucose determination

Food was removed overnight. Blood samples were taken by caudal vein puncture under ethyl ether anesthesia. Blood glucose levels were determined by CareSens blood glucose monitoring system (i-SENS, Seoul, Korea) according to the manufacturer’s instructions.

### Histological evaluation

Knees were dissected post-mortem, fixed in 10% formalized saline, decalcified, dehydrated and embedded in paraffin. Sections of 5 μm were made and stained with hematoxylin-eosin staining. Serial sections were evaluated by two blinded investigators. The inflammatory metacarpophalangeal joints were analyzed according to different parameters, including the influx of inflammatory cells in joint capsules, cartilage destruction, joint cavity narrowing, influx of inflammatory cells in marrow cavities and periosteal thickening.

All procedures involving animals were performed in accordance with the European Communities Council Directive of November 24, 1986 (86/609 EEC) and approved by the Ethics Committee of Changhai Hospital.

### Statistical analysis

All data were presented as the means ± standard deviation (S.D). Statistical significance was determined using SPSS 11.0 for Windows from SPSS Inc. Chicago, Illinois, USA. Data analysis was performed by one-way analysis of variance (ANOVA), followed by Fisher’s least significant difference (LSD). Differences with *P-*values *<*0.05 were considered to be statistically significant.

## Results

### Ginsenoside Rh1 combined with DEX inhibited the expression of pro-inflammatory cytokines, even after long-time treatment

Our recent research showed that ginsenoside Rh1 could reverse the reduction of GR expression and binding capacity induced by DEX (Additional file 1: Figure S1). And in the presence of 10 μM Rh1, the reversal effect was the most significant.

In order to observe if Rh1 could improve the anti-inflammatory potential of DEX, we compared the therapeutic potential of both treatment protocols (Rh1 combined with DEX and DEX treatment alone) in two different experimental settings. First, we pretreated the RAW264.7 cells with solvent, DEX (1 μM) or DEX combined with Rh1 (10 μM, 1 μM, 0.1 μM) or Rh1 alone for 2 h, after which TNF-α (20 ng/ml) was added for 8 h. The anti-inflammatory agents thus resided on the cells for a time span of 10 h, hereby mimicking a short-term treatment protocol. Alternatively, we pretreated the cells with solvent, DEX (1 μM) or DEX combined with Rh1 (10 μM, 1 μM, 0.1 μM ) or Rh1 alone for 24 h, after which TNF-α was added for 8 h. The anti-inflammatory agents were thus kept on the cells for 32 h, reflecting a prolonged treatment protocol.

**Figure 1:**
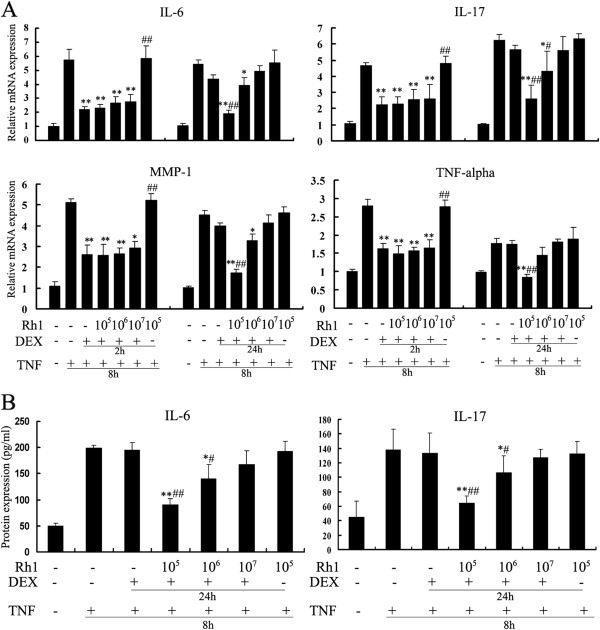
**Effects of ginsenoside Rh1 combined with DEX on TNF-induced cytokine production.** RAW264.7 cells were pretreated with solvent, DEX (1 μM) or DEX combined with Rh1 (10 μM, 1 μM, 0.1 μM ) or Rh1 alone for 2 h (short-term treatment protocol) or 24 h (prolonged treatment protocol), as indicated in the figures. Afterwards, TNF (20 ng /ml) was added for 8 h. **(A)** Expression of the pro-inflammatory mediators IL-6, IL-17, MMP-1 and TNF-α was monitored by means of quantitative PCR. Gene expression of the housekeeping gene *β-actin* was used for normalization. **(B)** Protein expression of IL-6 and IL-17 were also determined by ELISA. Statistical significance was determined by one-way analysis of variance (*P <0.05, **P <0.01 versus TNF group; #*P* <0.05, ##*P* <0.01 versus the DEX group). The experiments were replicated three times, and results are representative of at least three independent induction experiments. DEX, dexamethasone; IL, interleukin; MMP-1, matrix matalloproteinase-1; TNF, tumor necrosis factor.

The pro-inflammatory mediators IL-6, IL-17, MMP-1 and TNF-α were chosen to evaluate the anti-inflammatory potential. We observed that DEX alone was sufficient to down-regulate the TNF-α-induced gene expression of IL-6, IL-17, MMP-1 and TNF-α for a short treatment, but was insufficient after a prolonged treatment. Interestingly, Rh1 (10 μM) combined with DEX treatment, even after prolonged treatment, efficiently repressed the expression of IL-6, IL-17, MMP-1 and TNF-α (Figure 1A). Similar results were found on the protein expression of IL-6 and IL-17 (Figure 1B). It is worth noting that Rh1 treatment alone could not repress the TNF-induced cytokines. No obvious inhibition in cell viability was observed in any experimental settings (Additional file 2: Figure S2).

### Ginsenoside Rh1 increased DEX induced inactivation of NF-κB and activation of DUSP1

**Figure 2:**
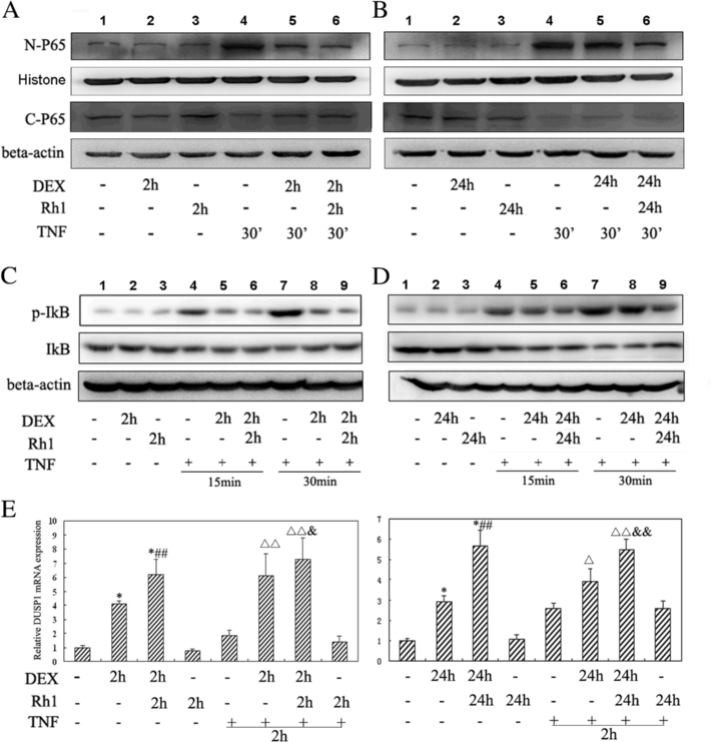
**Effects of ginsenoside Rh1 combined with DEX on TNF-induced NF-κB translocation and DUSP1 activation.** After pretreatment with solvent, DEX (1 μM), or Rh1 (10 μM) combined with DEX for 2 h or 24 h, **(A-B)** TNF (20 ng /ml) was added for 30 minutes and localization of p65 was determined by Western blot. **(C-D)** TNF was added for the indicated time periods (15 and 30 minutes) and expression of phospho-IκBα and total IκBα was determined. The experiment was replicated three times. **(E)** After pretreatment with solvent, DEX, Rh1 combined with DEX, or Rh1 for 2 h or 24 h, TNF was added for 2 h and DUSP1 was determined by means of quantitative PCR. Gene expression of the housekeeping gene *β-actin* was used for normalization. Statistical significance was determined by one-way analysis of variance (*P <0.01 versus Control; #*P* <0.05, ##*P* <0.01 versus DEX group; Δ*P* <0.05, ΔΔ*P* <0.01 versus TNF group; &*P* <0.05, &&*P* <0.01 versus TNF + DEX group). The experiments were replicated three times, and the results are representative of at least two independent induction experiments. DEX, dexamethasone; DUSP1, dual specificity protein phosphatase 1; TNF, tumor necrosis factor.

Because Rh1 could promote the anti-inflammatory potential of DEX after long-time treatment, we tested whether this inhibition is partly dependent on inactivation of NF-κB or activation of DUSP1. The data showed that DEX could efficiently prevent the translocation of p65 from cytoplasm to nucleus in a short treatment protocol (lane 5 *vs* 4 in Figure 2A) but not in a prolonged treatment protocol (lane 5 *vs* 4 in Figure 2B). In contrast, even after prolonged treatment, Rh1 combined with DEX was still able to efficiently inhibit the translocation of p65 (lane 6 *vs* 4 in Figure 2B). Furthermore, phospho-IκBα and total IκBα protein levels were assessed. In the short treatment protocol, either DEX alone or Rh1 combined with DEX could result in a decrease of phospho-IκBα (lane 5, 6 *vs* 4, lane 8, 9 *vs* 7 in Figure 2C). When administered for a prolonged time, DEX alone failed to inactivate IκBα (lanes 5 *vs* 4, lanes 8 *vs* lane 7 in Figure 2D). However, Rh1 combined with DEX was still able to dephosphorylate IκBα efficiently, even after prolonged treatment, which was accordant to the result of p65 (lane 6 *vs* lane 4, lane 9 *vs* lane7 in Figure 2D).

Another mechanism by which glucocorticoids inhibit inflammation is through induction of DUSP1. Figure 2E showed that either in short-term treatment or prolonged treatment protocol, DEX could increase the transcription of DUSP1 with or without TNF-α. It was worth noting that the degree of activation of DUSP1 by DEX in 24 h-treatment group is lower than those in the 2 h-treatment group. More important, Rh1 could enhance DEX-induced DUSP1 expression in both protocols. However, Rh1 alone had no effect on the transcription of DUSP1.

### Ginsenoside Rh1 potentiated DEX induced inactivation of p38

**Figure 3:**
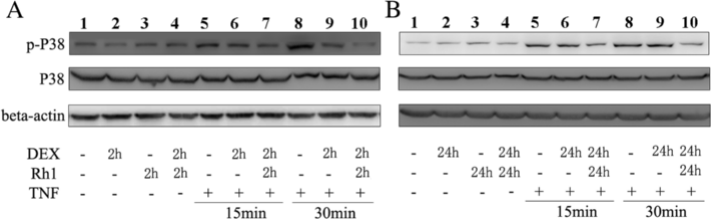
**Effects of ginsenoside Rh1 combined with DEX on p38 activation.** After pretreatment with solvent, DEX (1 μM), or Rh1 (10 μM) combined with DEX for 2 h or 24 h, TNF (20 ng /ml) was added for the indicated times (15 and 30 minutes) and expression of phospho-p38 and total p38 was determined by Western blot. The experiment was replicated three times, and the results are representative of at least two independent induction experiments. DEX, dexamethasone; TNF, tumor necrosis factor.

As MAPK phosphatase 1, DUSP1 can dephosphorylate p38, playing an important role in the stimulation of the inflammatory response and having detrimental effects on GR ligand binding. The effect of Rh1 on p38 MAPK was investigated for the increase of Rh1 on DEX-induced DUSP1. As expected, stimulation of RAW264.7 cells with TNF-α for 15 and 30 minutes resulted in significant increases in p38 activation (lanes 5 and 8 *vs* lane 1 to 4 in Figure 3A, B). DEX treatment alone inhibited TNF-α induced activation of p38 in a short treatment protocol (lanes 6 *vs* 5, lanes 9 *vs* 8 in Figure 3A), but not in a prolonged protocol (lanes 6 *vs* 5, lanes 9 *vs* 8 in Figure 3B). However, Rh1 combined with DEX was still able to dephosphorylate p38 efficiently even after prolonged treatment (lane 7 *vs* 5, lane 10 *vs* 8 in Figure 3B). These analyses were also quantified by ImageQuant (Additional file 3: Figure S3).

### Improvement of GR was responsible for ginsenoside Rh1 enhancing the anti-inflammatory potential of DEX after long-time treatment

**Figure 4:**
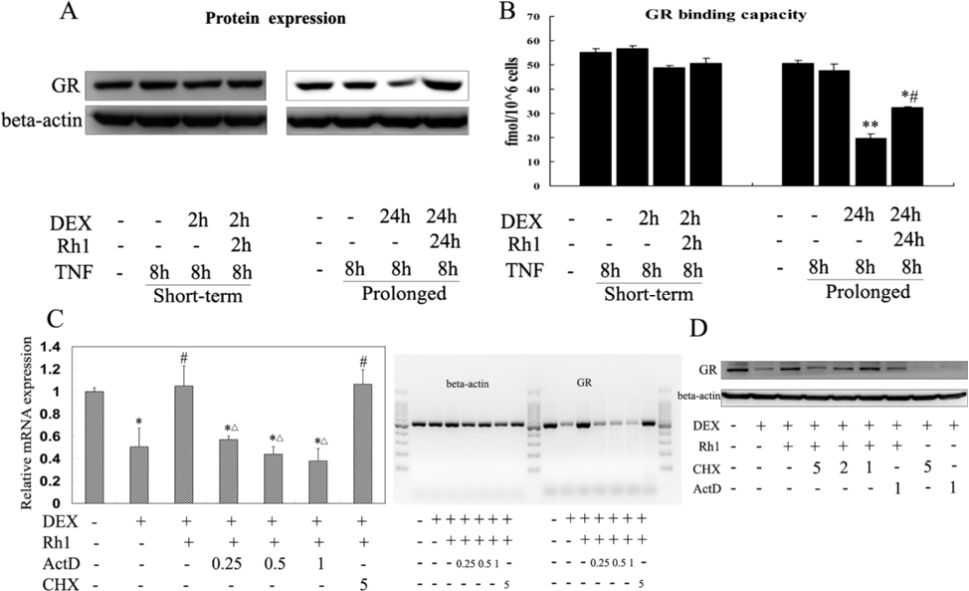
**Effects of ginsenoside Rh1 combined with DEX on GR. (A-B)** After pretreatment with solvent, DEX (1 μM) or Rh1 (10 μM) combined with DEX for 2 h or 24 h, TNF (20 ng/ml) was added for 8 h. **(A)** Western blot analysis was performed in parallel on total extracts with an anti-GR antibody. The detection of β-actin was used as a loading control. **(B)** Specific binding of GR was also determined in parallel on RAW 264.7 cells. RAW264.7 cells (1 × 10^6^) were incubated 3 h with 10^−6^ M [^3^H] DEX, with or without excess unlabeled DEX. Specific binding was determined. *P <0.05, **P <0.01 versus control, #*P* <0.01 versus DEX group. **(C-D)** RAW264.7 cells were treated with solvent, DEX alone or Rh1 combined with DEX for 24 h. **(C)** Transcription was then stopped by addition of actinomycin D (0.25, 0.5, 1 ng/ml) in the presence of Rh1. GR mRNA levels were assessed by means of quantitative PCR. β-actin was used for normalization. The left panel is the quantitative result and the right panel is RNA electrophoretic figures. *P <0. 01 versus control; #*P* <0.01 versus DEX group; Δ*P* <0.01 versus Rh1 + DEX group. **(D)** Protein synthesis was stopped by addition of cycloheximide (5, 2, 1 μg/ml) in the presence of Rh1. GR levels were determined by Western blot. The detection of β-actin was used as a loading control. The experiment was replicated three times and results are representative of at least two independent induction experiments. DEX, dexamethasone; GR, glucocorticoid receptor; TNF, tumor necrosis factor.

To observe the correlation between the GR and anti-inflammatory potential of Rh1 combined with DEX, a parallel Western blot experiment and saturation binding analysis were performed. The results confirmed that, in the prolonged treatment protocol, DEX-induced down-regulation of the GR expression and binding capacity became more pronounced than those in the short-term treatment protocol, thereby explaining the abolishment of its anti-inflammatory effects. However, Rh1 combined with DEX could partially reverse the decreased GR expression and binding affinity in RAW 264.7 cells, and this was reflected by its efficiency in anti-inflammation, even on a long-term basis as described above (Figure 4A, B). Furthermore, co-treatment of Rh1 with an inhibitor of mRNA transcription, actinomycin-D (0.25, 0.5, 1 ng/ml) or with an inhibitor of protein synthesis, cycloheximide (5, 2, 1 μg/ml), abolished the Rh1-induced up-regulation of GR (Figure 4C, D), suggesting that mRNA transcription and the new protein synthesis were both involved in the effect of Rh1 on GR.

### Ginsenoside Rh1 exhibited a little promotion on GRE promotor activity but played different roles on GRE-driven genes expression

**Figure 5:**
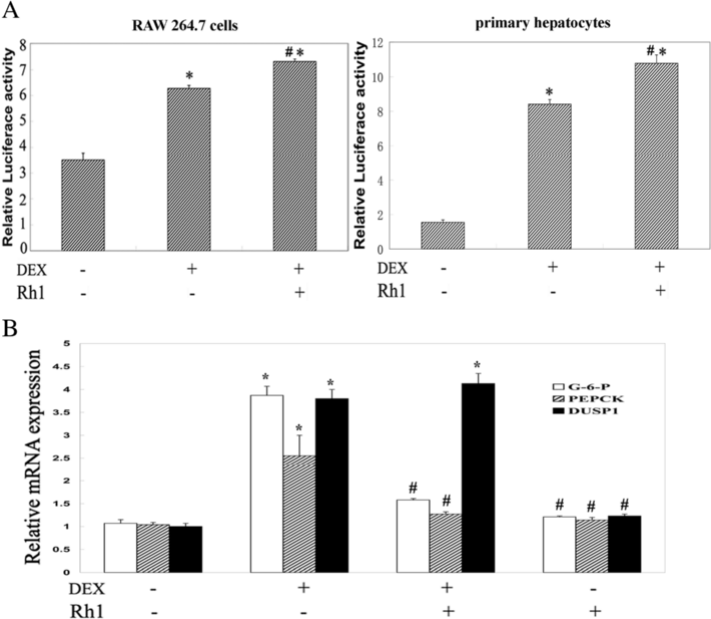
**Effect of Rh1 combined with DEX on GRE activity and some gluconeogenesis-related genes.** RAW264.7 cells were treated with solvent, DEX alone or Rh1 combined with DEX for 24 h. **(A)** Cell lysates were assayed for luciferase activities. Promoter activities are expressed as relative induction factor, that is, the ratio of expression levels recorded either at induced and non-induced conditions, with the latter taken to be 1. Assays were performed in triplicate, and results are representative of at least two independent induction experiments. **(B)** Primary hepatocytes were treated with solvent, DEX alone or Rh1 combined with DEX for 24 h. RNA was isolated and reverse transcribed. The resulting cDNA was subjected to PCR analysis with primers to detect the household gene GAPDH (loading control) or the gene coding for DUSP1, PEPCK and G6P in the same sample. *P <0.01 versus control, #*P* <0.01 versus DEX group. DEX, dexamethasone; DUSP1, dual specificity protein phosphatase 1; G6P, glucose-6-phosphatase; GRE, glucocorticoid receptor elements; PEPCK, phosphoenolpyruvate carboxykinasee phosphatase; TNF, tumor necrosis factor.

For GR up-regulation being associated with enhancement of GRE promoter activity, we evaluate the GRE promoter activity by luciferase assay analysis. It seems that Rh1 could enhance the DEX-induced GRE promoter activity slightly in RAW264.7 cells and the primary mouse hepatocytes (Figure 5A). Interestingly, although Rh1 could enhance DEX induced DUSP1 expression, Rh1 treatment alone or Rh1 combined with DEX could not enhance the expression of PEPCK or G6P in primary mouse hepatocytes, which was different from DEX. It seems that Rh1 could inhibit the expression of PEPCK or G6P in the hepatocytes which stimulated by DEX (Figure 5B).

### Ginsenoside Rh1 combined with DEX markedly attenuated inflammation in CIA model

**Figure 6:**
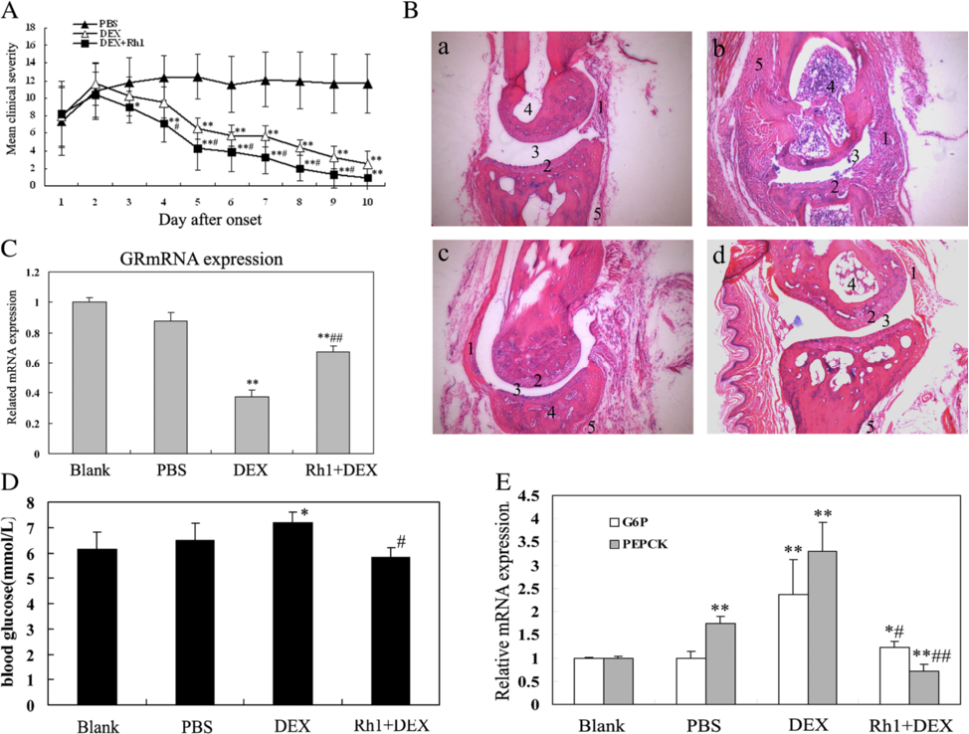
**Anti-inflammatory potential of ginsenoside Rh1 combined DEX in CIA. (A)** After onset of arthritis, CIA mice were randomized in a PBS, DEX (1 mg/kg) treatment or Rh1 (10 mg/kg) combined with DEX (1 mg/kg) treatment protocol. Disease severity was monitored daily. Dots and bars represent mean ± SEM. *P <0.05, **P <0.01 compared with PBS, # *P* <0.05 compared with DEX. **(B)** Histopathology of paw joints 10 days post-arthritis onset. HE staining of the metacarpophalangeal joint: **a**, **b**, **c** and **d** are representative images for blank (normal mice), PBS-, DEX- and Rh1 combined with DEX-treated mice, respectively. The different parameters assessed are 1) influx of inflammatory cells in joint capsule, 2) cartilage destruction, 3) joint cavity narrowing, 4) influx of inflammatory cells in marrow cavities, and 5) and periosteal thickening. **(C)** Real time PCR was performed in parallel on liver tissues extracts with GR primers. Gene expression of the housekeeping gene *GAPDH* was used for normalization. *P <0.05, **P <0.01 compared with blank, ##*P* <0.01 compared with DEX. **(D)** The blood glucose concentration was determined 6 h after treatment (mice were fasted 18 h before the blood samples were taken). The normal mice were as blank. Values are expressed as means ± SD. *P <0.01 versus blank??; #*P* <0.01 versus DEX group. **(E)** Liver samples were taken from the CIA mice after 10 days of treatment. PCR analysis with primers detects the household gene *β-actin* (loading control) or the gene coding for PEPCK and G6P in the same sample. *P <0.05, **P <0.01 compared with blank, #*P* <0.05, ##*P* <0.01 compared with DEX. CIA, collagen-induced arthritis; DEX, dexamethasone; G6P, glucose-6-phosphatase; GR, glucocorticoid receptor; PBS, phosphate-buffered saline; PEPCK, phosphoenolpyruvate carboxykinasee phosphatase.

To evaluate the *in vivo* potency of Rh1 combined with DEX to suppress Ag-driven immune responses, we examined its effect in CIA. No differences either in the mean severity or in the time to onset of arthritis could be observed. Although the DEX-treated mice showed a steady decrease in clinical severity over time, more notable anti-inflammatory responses in the Rh1 combined with DEX-treated mice were observed, as compared with DEX treatment alone. Either in the mean anti-inflammatory efficiency in every day or in the time to onset of anti-inflammation, Rh1 combined with DEX-treatment showed a better superiority of anti-inflammatory potential than DEX treatment alone (Figure 6A).

After clinical assessment for 10 days, mice were sacrificed and paw joints were histologically examined to determine whether the amelioration of clinical disease activity was accompanied with reduced histopathological signs of inflammation and tissue damage. The majority of joints from vehicle-treated animals showed evidence of the influx of inflammatory cells in marrow cavities and joint capsules, destruction of articular cartilage and inflammation resulting in periosteal thickening (Figure 6B-b). In this analysis, only 30% of the joints from the DEX-treated mice were found to be normal in all assessed parameters, which still showed inflammatory cells in marrow cavities and joint capsules, and periosteum thickening (Figure 6B-c). However, Rh1 combined with DEX treatment group was accompanied by normal histopathologic features in all animals. The effects were most apparent with regard to the reduction in the degree of inflammation in the joint capsules and periosteum (Figure 6B-d).

A parallel Real-time PCR for GR mRNA was performed. GR mRNA after DEX treatment was significantly lower than those after Rh1 combined with DEX treatment (*P* <0.01), and this was reflected by its efficiency in anti-inflammation in CIA as described *in vitro* (Figure 6C).

Additionally, effects of Rh1 on blood glucose were also observed *in vivo*. High blood glucose levels were found in the DEX treatment group (*P* <0.01) but not in the Rh1 combined with DEX group (*P* >0.05, Figure 6D). Furthermore, by RT-PCR analysis, Rh1 could ameliorate DEX-induced high expression of the PEPCK or the G-6-P in CIA (*P* <0.05, Figure 6E). It seemed that Rh1 combined with DEX had fewer negative effects than DEX treatment alone on blood glucose *in vivo*.

## Discussion

The present study demonstrates some intriguing molecular aspects of glucocorticoid receptor biology, which have to be kept in mind when developing new strategies to reduce glucocorticoid resistance and related side effects. The results showed that ginsenoside Rh1 could improve the anti-inflammatory potential of DEX even after prolonged treatment. Most interestingly, Rh1 could ameliorate DEX- induced hyperglycemia *in vitro* and *in vivo*.

The mechanisms linked to the phenomenon of glucocorticoid resistance are complex [4,24]. Several therapeutic strategies for the management of glucocorticoid-resistant diseases have been proposed, but most important approaches involve improving anti-inflammatory potential of glucocorticoids to reduce treatment time or reversing the molecular mechanisms of glucocorticoid resistance. Some selective glucocorticoid receptor agonists (SEGRAs; or dissociated steroids) may exert their anti-inflammation without provoking ligand-induced resistance [9]. However, it is unlikely that SEGRAs will be able to overcome glucocorticoid resistance because they might be as ineffective as conventional glucocorticoids in triggering gene activation or repression [25]. Alternative anti-inflammatory drugs are currently available for the treatment of glucocorticoid-resistant diseases, but these drugs are likely to have issues with toxicity and side-effects, or are only suitable for topical application [26].

The results presented in this study showed that RAW 264.7 cells were only initially responsive to the therapeutic effects of DEX after a short-term treatment, but for a prolonged time, the cells became insensitive to DEX. When the cells were treated with DEX combined with Rh1, the anti-inflammatory potential of DEX was preserved in both short and prolonged usage. Rh1 itself had no effects on TNF-induced cytokines, so the heightened anti-inflammatory effect of Rh1 combined with DEX may be attributed to Rh1’s improvement on GC-GR interaction. In fact, GCs exhibit potent anti-inflammatory effects through two main mechanisms. First, they inhibit the transcription of pro-inflammatory genes via suppression of the transcriptional activation induced by AP-1 and NFκB. Second, they induce genes that antagonize the inflammatory response, including the glucocorticoid-induced leucine zipper (*GILZ*) and *DUSP1*[26]. Our data showed that in contrast to DEX, Rh1 combined with DEX still could attenuate p65 cytoplasmic-to-nuclear translocation after prolonged treatment, which is a prerequisite for activation of NFκB, by decreasing the phosphorylation of IκBα and attenuating IκBα degradation. Also, Rh1 combined with DEX induced a higher expression of DUSP1 than DEX alone in either short-term treatment or prolonged treatment. DUSP1 is a member of a large family of multifunctional phosphatases that reside in the nucleus and specifically dephosphorylate and inactivate members of the MAPK family, such as JNK, p38 MAPK and ERK [4,24]. Increased expression of DUSP1 attenuates p38 MAPK signaling, which disrupts the signal leading to the induction of pro-inflammatory gene expression. In this report, we demonstrated that dephosphorylation of p38 declined after prolonged treatment with DEX. But Rh1 combined with DEX resulted in striking inhibition of p38 activation even after prolonged usage, which is accordant to the DUSP1 result. These findings together show that Rh1 can reinforce the anti-inflammatory potential of DEX and alleviate GC resistance induced by prolonged DEX treatment. More importantly, this improvement was a result of both transrepression and transactivation of GR. Since down-regulation of GR is associated with the loss of GCs’ responsiveness, this aspect was also examined in our study. The results indicated that Rh1 could ameliorate DEX-induced down-regulation of GR expression and binding, in accordance with the retained anti-inflammatory effects even after prolonged treatment. This is in contrast to DEX alone, showing a direct link between prolonged treatment, decreasing GR and the abolishment of the anti-inflammatory effects. Furthermore, as Rh1-induced up-regulation of GR was blocked by the co-treatment with actinomycin-D or cycloheximide, *de novo* mRNA transcription and protein synthesis are most likely involved. These findings suggested that long-term Rh1 treatment might not only increase the transcription of GR, but also its synthesis in RAW macrophages. And Rh1’s up-regulated expression of GR and dephosphorylation of p38MAPK might be responsible for its improvement on GR ligand binding. It was reported that activated p38 targeted the GR ligand-binding domain indirectly to suppress GR function [27]. Therefore, inhibition of p38 phosphorylation by Rh1 may benefit GR ligand binding. The role of p38 in the mechanism of action of Rh1 on GR ligand binding is therefore worthy of further study.

Although Rh1 could increase DEX-induced GRE activation and DUSP1 expression, Rh1 combined with DEX did not induce high expression of G-6-P and PEPCK in primary hepatocytes from mice, which is important in gluconeogenesis. It was interesting that Rh1 played different roles on GRE-driven genes. Therefore, the structural changes of GR after Rh1 treatment, as well as some transcriptional factors involved require further study. Altogether, because the most attractive option for treating glucocorticoid resistance is to directly address the cause, Rh1 combined with DEX may be an effective treatment protocol for acquired glucocorticoid resistance with down-regulation of GR protein levels and binding.

To confirm our *in vitro* data, we examined the role of Rh1 combined with DEX in the CIA model. It seems that Rh1 is able to improve the anti-inflammatory effects of DEX *in vivo*. In line with this, improved GR expression was found in liver isolated from mice treated with Rh1 combined with DEX. Meanwhile, DEX combined with Rh1 had no unwanted effects on blood glucose levels, in sharp contrast with the effect of DEX treatment alone. Further analysis of G6P and PEPCK GRE-driven gene expression in the liver demonstrated the beneficial regulatory effect of Rh1 on glucose metabolism. Although the CIA model did not mimic GC-resistance *in vivo*, our data still supported that Rh1 increased the anti-inflammatory potential of DEX without hyperglycemia and improved DEX-induced down-regulation of GR *in vivo*.

## Conclusions

Ginsenoside Rh1 potentiated DEX’s anti-inflammatory effects by improving DEX-induced down-regulation of GR. Our data also showed that ginsenoside Rh1 combined with DEX did not cause hyperglycemia. As a result, ginsenoside Rh1 combined with DEX may be a promising candidate treatment option for chronic inflammatory diseases that require long-term immunosuppression therapies and so is worthy of study.

## Abbreviations

CIA: Collagen-induced arthritis; DEX: dexamethasone; DMEM: Dulbecco’s modified Eagle’s medium; DUSP1: dual specificity protein phosphatase 1; FBS: fetal bovine serum; G6P: gluconeogenic enzyme glucose-6-phosphatase; GAPDH: glyceraldehyde 3-phosphate dehydrogenase; GCs: glucocorticoids; GILZ: glucocorticoid-induced leucine zipper; GR: glucocorticoid receptor; GRE: glucocorticoid-responsive elements; GSS: ginsenosides; IL-17: Interleukin-17; IL-6: Interleukin-6; MMP-1: matrix matalloproteinase-1; NF-κB: nuclear factor kappa B; PBS: phosphate-buffered saline; PEPCK: phosphoenolpyruvate carboxykinasee phosphatase; SEGRAs: selective glucocorticoid receptor agonists; TNF-α: tumor necrosis factor-alpha.

## Competing interests

The authors declare that they have no competing interests.

## Authors’ contributions

JL was involved in data collection and analysis, manuscript writing, critical revision and final approval of the manuscript. JD was responsible for conception and design of the study, data collection and analysis, manuscript writing and final approval of the manuscript. DL, BBC, FFF and LW were responsible for data collection, critical revision and final approval of the manuscript. CW contributed to the design of the study, data analysis, critical revision and final approval of the manuscript. CQL contributed to the conception and design of the study, financial support, critical revision and final approval of the manuscript. All authors read and approved the final manuscript.

## Authors’ information

Jun Li and Juan Du are listed equally as first authors.

## Supplementary Material

Additional file 1: Figure S1 Effects of ginsenoside Rh1 on glucocorticoid receptor (GR). RAW 264.7 cells were treated with solvent (DMSO), DEX (1 μM), or Rh1 (10 μM, 1 μM and 0.1 μM as indicated in the figures) for 1 h followed by DEX, for 24 h. (A) Western blot analysis was performed on total protein extracts with an anti-GR antibody. The detection of β-actin was used as a loading control. (B) Saturation binding analysis of GR on RAW264.7 cells. RAW264.7 cells (1×10^6^) were incubated 3 h with 10^-6^ M [^3^H] DEX, with or without excess unlabeled Dex. Specific binding was determined. *P <0.01 versus Control, #*P* <0.05, ##*P* <0.01 versus DEX group. Click here for file

Additional file 2: Figure S2 Rh1 combined with has little cytotoxicity on RAW264.7 cells. RAW264.7 cells (1 × 10^4^ cells) were pretreated with either DEX alone or in the presence of 0.1 to 10 μM Rh1 for 24, after that TNF (20 ng/ml) was added for 8 h. At the end of incubation, cell number was examined by MTT method. This experiment is representative of three independent experiments. Click here for file

Additional file 3: Figure S3 Effects of ginsenoside Rh1 combined with DEX on p38 activation. After pretreatment with solvent, DEX (1 μM) or Rh1 (10 μM) combined with DEX for 2 h or 24 h, TNF (20 ng/ml) was added for the indicated times (15 and 30 minutes) and expression of phospho-p38 and total p38 was determined by Western blot. The result was quantified by ImageQuant 5.2. The experiment was replicated three times, and the result is the average of three experiments. *P <0.01 versus blank group; #*P* <0.01 versus TNF group. Click here for file
