# Supplementary figures and images for: Ginsenoside Rh1 potentiates dexamethasone’s anti-inflammatory effects for chronic inflammatory disease by reversing dexamethasone-induced resistance
Source: Arthritis Res Ther. 2014 May 1;16(3):R106. doi: 10.1186/ar4556 (PMC4060561; doi:10.1186/ar4556)

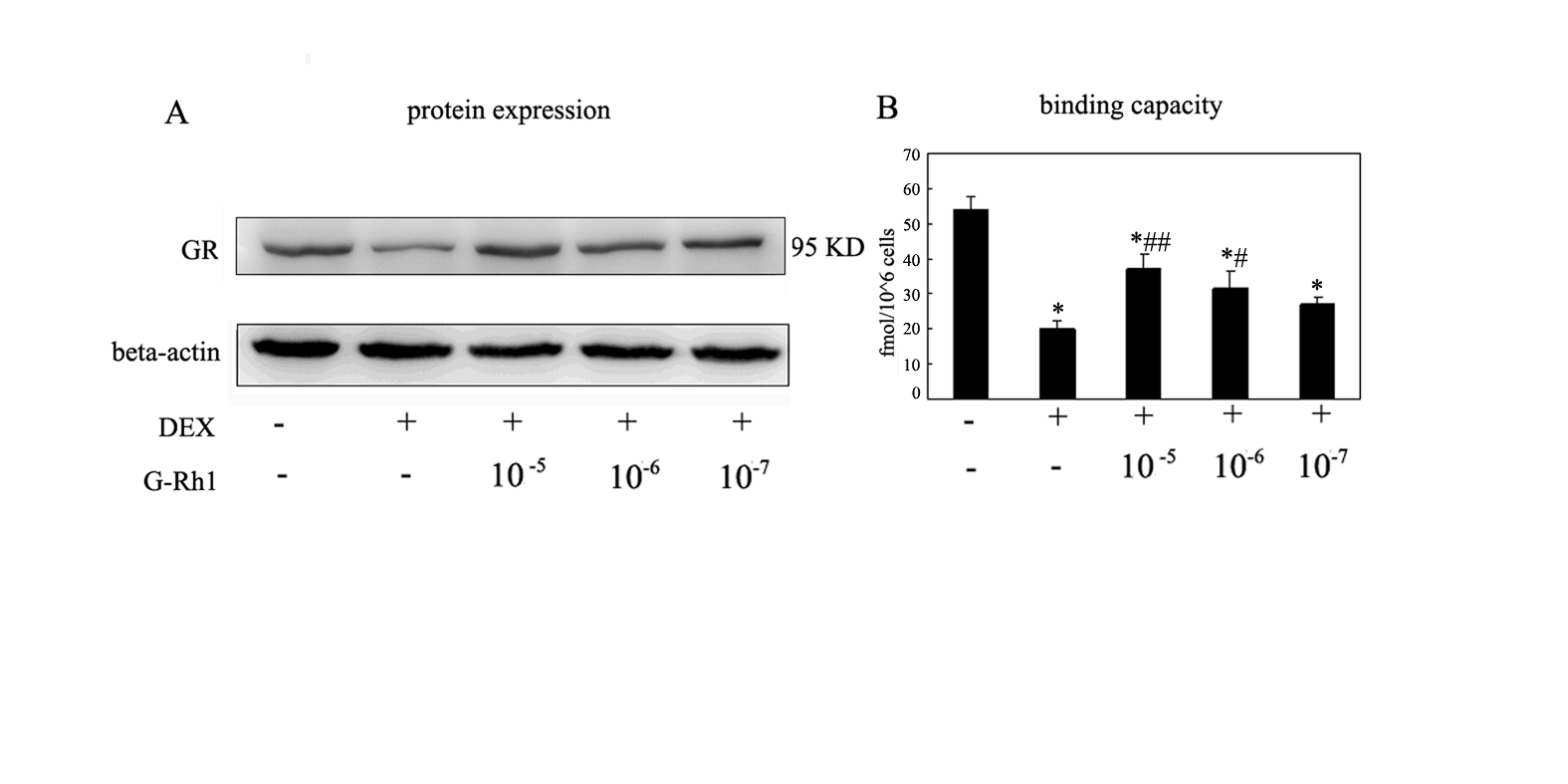

Supplement: Additional file 1: Figure S1 — Effects of ginsenoside Rh1 on glucocorticoid receptor (GR). RAW 264.7 cells were treated with solvent (DMSO), DEX (1 μM), or Rh1 (10 μM, 1 μM and 0.1 μM as indicated in the figures) for 1 h followed by DEX, for 24 h. (A) Western blot analysis was performed on total protein extracts with an anti-GR antibody. The detection of β-actin was used as a loading control. (B) Saturation binding analysis of GR on RAW264.7 cells. RAW264.7 cells (1×10^6^) were incubated 3 h with 10-6 M [3H] DEX, with or without excess unlabeled Dex. Specific binding was determined. *P <0.01 versus Control, #P <0.05, ##P <0.01 versus DEX group. [file ar4556-S1.tiff]

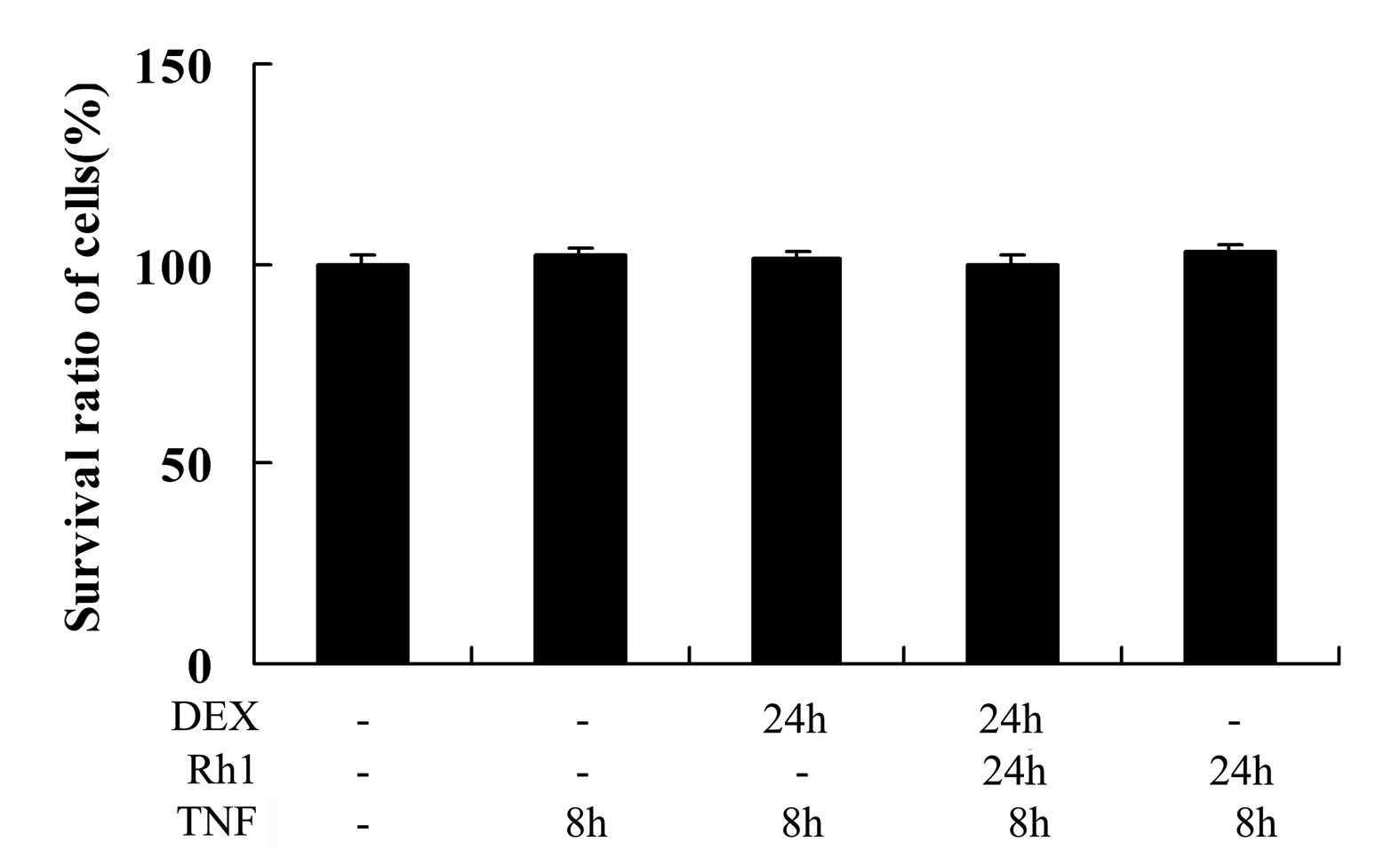

Supplement: Additional file 2: Figure S2 — Rh1 combined with has little cytotoxicity on RAW264.7 cells. RAW264.7 cells (1 × 10^4^ cells) were pretreated with either DEX alone or in the presence of 0.1 to 10 μM Rh1 for 24, after that TNF (20 ng/ml) was added for 8 h. At the end of incubation, cell number was examined by MTT method. This experiment is representative of three independent experiments. [file ar4556-S2.tiff]

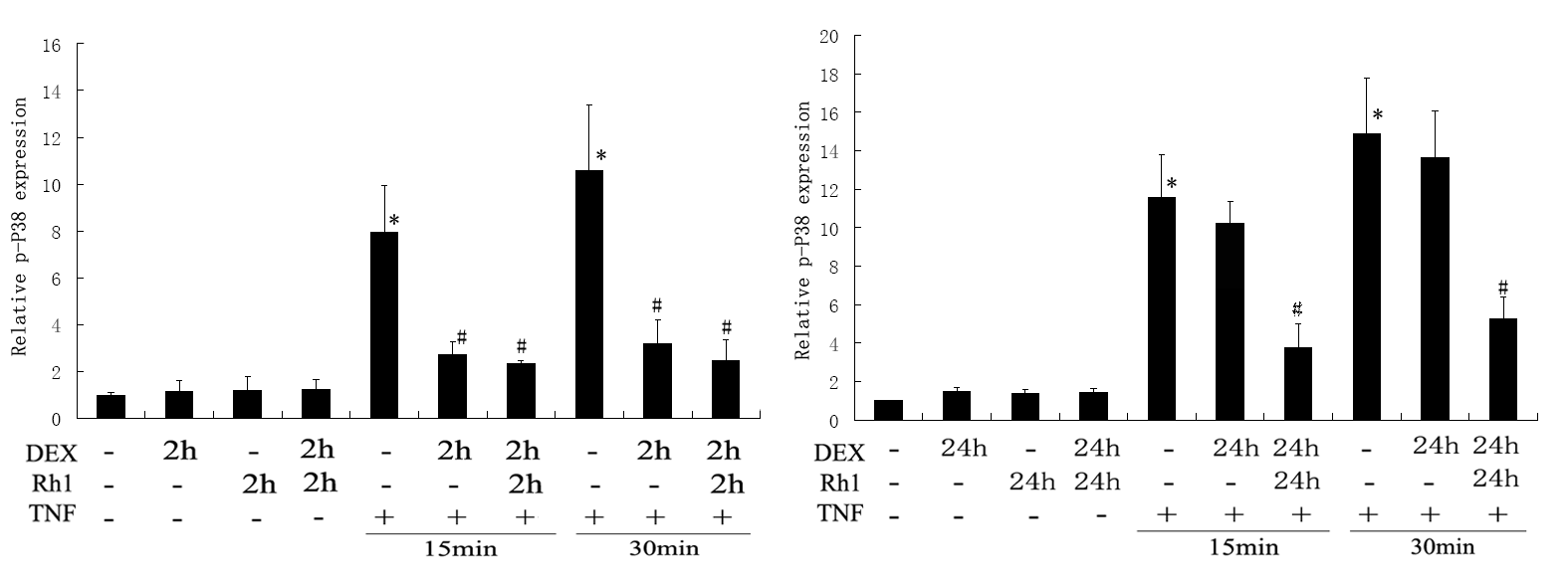

Supplement: Additional file 3: Figure S3 — Effects of ginsenoside Rh1 combined with DEX on p38 activation. After pretreatment with solvent, DEX (1 μM) or Rh1 (10 μM) combined with DEX for 2 h or 24 h, TNF (20 ng/ml) was added for the indicated times (15 and 30 minutes) and expression of phospho-p38 and total p38 was determined by Western blot. The result was quantified by ImageQuant 5.2. The experiment was replicated three times, and the result is the average of three experiments. *P <0.01 versus blank group; #P <0.01 versus TNF group. [file ar4556-S3.tiff]
